# Adjacent Fracture Rates Following Balloon Kyphoplasty in Osteoporotic Vertebral Compression Fractures: A Case Series

**DOI:** 10.7759/cureus.40651

**Published:** 2023-06-19

**Authors:** Hannah Goldman-Daleo, Benjamin Rachman, Rahul Mhaskar

**Affiliations:** 1 Interventional Radiology, University of South Florida Morsani College of Medicine, Tampa, USA; 2 Internal Medicine, University of South Florida Morsani College of Medicine, Tampa, USA

**Keywords:** kyphoplasty, adjacent fracture, osteoporosis, compression fracture, vertebroplasty, vertebral augmentation, spine, kyphoplasty pain, refracture

## Abstract

Background

Osteoporotic vertebral compression fractures (OVCF) represent a substantial concern, as they are associated with significant increases in morbidity and mortality. One option for the management of these patients is balloon kyphoplasty, in which a balloon is inflated within the collapsed vertebral body. Following this, the cavity is filled with polymethyl methacrylate (PMMA) cement to restore height and strength. Although this procedure has been shown to have great effectiveness, one complication that has been documented is an adjacent level refracture. This is thought to be due to the increased relative strength of the repaired vertebral body. Our study aims to quantify the rates of adjacent level refracture following balloon kyphoplasty as well as identify factors that may be associated with this event.

Methods

We reviewed the electronic medical records (EMR) of patients that underwent balloon kyphoplasty between January 1, 2017 and August 1, 2020. A single surgeon performed all procedures. Only adult patients who received a diagnosis of osteoporosis based on a history of fragility fracture or bone mineral density measurement were included. Patients with additional or confounding bone conditions, such as malignancy or other lytic lesions, were excluded. Data were analyzed in SPSS (IBM Corp., Armonk, NY, United States).

Results

We included 89 patients in our study. We observed an adjacent level refracture prevalence of 13.5% (n=12). We observed a significant increase in refracture rates among patients with unsatisfactory resolution of symptoms following initial balloon kyphoplasty, from 8.2% for satisfactory resolution of symptoms to 43.8% for those with unsatisfactory resolution. (p-value 0.011). Additionally, all 12 patients with adjacent level refractures occurred among patients with complex, multiple-level initial fracture patterns.

Conclusions

The treatment of OVCF with balloon kyphoplasty is a well-documented and effective method. The prevalence of adjacent-level refracture may be linked to several variables such as the initial fracture pattern. More research is needed to better predict refracture and improve patient outcomes.

## Introduction

Osteoporotic vertebral compression fractures (OVCF) represent a significant burden of morbidity and mortality in developed nations as shown in a 2006 study as measured by the disability-adjusted life years (DALYs) associated [1]. The total number of associated DALYs lost was 5.8 million, of which 51% were in Europe and the Americas. Notably, the DALYs lost due to osteoporotic fractures were higher than most cancers, with lung cancer being a notable exception [1]. Height restoration is a key factor in improving outcomes and has been shown to reduce post-fracture kyphosis, pulmonary-related mortality, and incidence of adjacent fracture in comparison to non-invasive management alone [2]. After failing medical management alone, balloon kyphoplasty can be used to achieve height restoration. Balloon kyphoplasty has been shown to have lower mortality, better pain reduction, and fewer complications than open correction [3]. In this procedure a balloon is inserted into a vertebral body under fluoroscopic guidance through the pedicle, inflated to restore the height of the vertebral body, and then removed. The now-existent cavity is filled with either polymethyl methacrylate or a cement intended to serve as a bone substitute [3]. Studies have measured the improvement in pain scores from the procedure as well as improvements in ambulation. These found a decrease in reported pain from 15/20 (visual analog scale) prior to surgery to 6/20 up to seven days post-surgery and an improvement in ambulation based on qualitative patient response in 49%-90% of patients who received either kyphoplasty or vertebroplasty [2].

Although this procedure has been found to be of considerably low risk and to have great effectiveness when compared with non-surgical management a significant post-operative concern of clinicians is fracture of an adjacent vertebral body in the months following the procedure [4]. One of the leading theories on why this occurs is due to increased stiffness of the operative vertebra causing increased strain on adjacent vertebrae. The incidence of subsequent fracture of an adjacent vertebral body has generally been demonstrated to be around 10%-10.5% [5,6]. Additionally, it has been shown that there is no statistically significant difference in rates of fracture of adjacent vertebral bodies in those who undergo balloon kyphoplasty as compared to those who are treated non-surgically [7].

The most important risk factor for subsequent fracture has been identified as cement leakage into the disk space, resulting in increased mechanical pressure and injury to the end plate in adjacent vertebral bodies. Leakage can be minimized when proper surgical techniques, including correct balloon placement, high-viscosity PMMA cement, controlled application, and limited volume injected, are employed [8].

## Materials and methods

The study was reviewed by the University of South Florida Institutional Review Board (STUDY001229) and a waiver of HIPAA authorization was granted. Patient medical records were searched for a single surgeon at an urban center to identify all patients that underwent balloon kyphoplasty for vertebral compression fracture from January 1, 2017 to August 1, 2020. Additional inclusion criteria included age over 18 years and a diagnosis of osteoporosis. Patients were excluded if additional diagnoses were present as a likely cause of vertebral compression fractures, such as malignancy with bony metastasis or trauma. Patient demographics included sex, BMI, age, height, weight, and ASA score. Other variables included subjective resolution of patient symptoms at the one-month post-operative follow-up, T-score where available, initial compression fracture location (defined as single lumbar, single thoracic, or multi-level involvement), and whether the patient was under medical therapy (including bisphosphonates, calcium, vitamin D supplements, or denosumab). The primary variable of interest was the presence of subsequent adjacent vertebral fractures. We conducted all analyses using IBM SPSS Statistics for Windows, Version 26.0 (Released 2019; IBM Corp., Armonk, United States). IBM SPSS Statistics for Windows, Version 26.0 (Released 2019; IBM Corp., Armonk, NY, United States). Either Fischer’s exact or Chi-squared test was used when appropriate.

## Results

One hundred ten patients were identified who received balloon kyphoplasty for vertebral compression fracture within the specified date range. 15 cases were excluded with trauma identified as the primary cause of fracture. An additional five cases were excluded to the presence of lytic bone lesions related to metastasis, and one further case was excluded due to insufficient follow-up of less than one month. Ultimately, 89 patients were included for analysis (Table 1). Our sample included 78 females and 11 males. The mean BMI was 24.7 (σ = 4.6), mean age 79.7 (σ = 9.1), mean weight 65.7 kg (σ = 13.7 kg). The mean T-score was -2.7 (σ = 0.7); however, this information was only available for 40 patients and therefore was not included in further analysis. All patients had an ASA score of 3.

**Table 1:** Sample characteristics Values given are mean (SD), apart from sex.

Patient characteristics (n=89)​	
Age​	79.7 (9.1)​
Sex (females)​	78​
BMI​	24.7 (4.6)​
Height (inches)​	64.2 (3.8)​
Weight (kgs)​	65.7 (13.7)​

Forty-eight patients in the sample had single lumbar fractures requiring initial repair. Twenty had single thoracic vertebral fractures. The remaining 21 had complex fracture patterns involving multiple levels and vertebrae. Eleven of 89 patients were on bisphosphonate therapy, 42 were using calcium supplements, 60 were using vitamin D supplements, and five were using denosumab. Sixty-six patients reported satisfactory resolution of fracture pain at one month follow up, while 23 continued to have residual pain that limited activity. Finally, 12 patients experienced an adjacent vertebral fracture (AVF) following initial balloon kyphoplasty, while 77 did not.

There was a significantly higher rate of fractures among men who received balloon kyphoplasty compared to women, 36.4% vs 10.3% (p = 0.038) (Table 2). There was an observed increase in the rate of fracture among patients who did not report satisfactory resolution of symptoms at one month, 21.7% vs 9.2%, though this was not statistically significant (p = 0.147). The initial fracture location (thoracic, lumbar, and multi-level) had no significant relationship with AVF (p = 0.305). There was a higher percentage of AVF in patients not taking bisphosphonates, with 12 fractures among those not taking bisphosphonates vs 0 who were, though this was not found to be significant (p = 0.348).

**Table 2:** Patient characteristics in relation to occurrence of adjacent level refracture. Fisher’s exact or the Chi-squared test was used where appropriate.​

Adjacent vertebral refracture characteristics​	Refracture (n = 12)​	No Refracture (n = 77)​	P-value​
Sex​	​	​	0.038​
Female​	8	70	​
Male​	4	7	​
Symptoms resolved at one month​	​	​	0.147​
Yes​	6​	59​	​
No​	5​	18​	​
Fracture Type​	​	​	0.305​
Thoracic​	4​	44​	​
Lumbar​	4​	16​	​
Multiple Level​	4​	17​	​
Bisphosphonates​	​	​	0.348​
Yes​	0​	11​	​
No​	12​	66​	​
Calcium Supplement​	​	​	0.030​
Yes​	2​	40​	​
No​	10​	37​	​
Vitamin D Supplement​	​	​	0.052​
Yes​	5​	55​	​
No​	7​	22​	​
Denosumab​	​	​	1.000​
Yes​	0​	5​	​
No​	12​	72​	​

## Discussion

Vertebral augmentation procedures are an important tool in the treatment of OVCF for patients who fail more conservative treatment options. Although similar, vertebroplasty and balloon kyphoplasty carry their own individual profiles for risks and complications. Vertebroplasty has more data analyzing these risks compared to balloon kyphoplasty. There is limited randomized controlled data investigating balloon kyphoplasty, and no sham trials currently. The majority of data in this realm stems from smaller retrospective studies and case series.

The largest randomized trial examining the incidence of adjacent fracture following BK included 151 patients in a BK group and 149 patients in a non-surgical group. Over 24 months of follow-up, 28 of 118 patients in the surgical group developed AVF, compared to 17 of 102. This difference was not statistically significant [4].

A meta-analysis of randomized controlled trials published in 2014 included six trials with a total of 424 patients, including studies until 2017. They observed a rate of AVF of 10.3%, similar to our own results. Additionally, this study had a maximum follow-up period was two years, with an average follow-up time of 10.7 months. Despite this longer follow-up period compared to our own results, there was a lower incidence of AVF [9]. In a second meta-analysis published in 2018, 12 studies were included with a total of 1951 patients. Studies were included up to 2014. This analysis, similarly, did not detect a significant difference in AVF incidence between BK and conservative management [7].

Since these prior meta-analyses were published, there have been several newer studies, mainly retrospective case series. They mostly support the position that BK is not associated with a higher incidence of AVF as compared to non-surgical treatment. They also suggest several common pre-operative variables that may be useful predictors for AVF. For example, in several studies, increased local kyphotic angle prior to surgery was significantly associated with fracture [10,11]. In other studies, they identify intra and postoperative variables that were associated with AVF, including overcorrection of vertebral height loss [12] and radiographic cement leakage [13,14].

Two smaller studies developed scoring systems incorporating several variables. The first included patient age, number of previous vertebral fractures, and local kyphosis among a sample of 65 patients with an overall incidence of AVF of approximately 30%. This paper obtained a correlation coefficient of 0.812 with a p-value of 0.05 [15]. In the second study, 109 patients undergoing BK with at least six months of follow-up were included, with 32 (29%) sustaining AVF. Predictive variables with statistically significant associations were found to be an increased wedge angle before BK, an increased degree of correction, and an initial fracture at T11-L2. Scores ranged from 0 to 6 in this system, with 100% of patients scoring 5-6 (n = 16) sustaining AVF [16].

This study’s results did not identify age as significantly associated with AVF, unlike the previously mentioned studies. Radiographic data were unavailable and our results cannot be compared to existing data on an initial kyphotic angle or post-operative cement leakage. Interestingly, there is very little data available on the association of AVF with standard medical therapies for osteoporosis. In this sample, calcium supplementation was significantly associated with decreased rates of AVF, with an odds ratio of 0.2 (p = 0.03). While the association with vitamin D was not significant (p = 0.052), this may be an issue of sample size. Bisphosphonate and Denosumab therapy were not associated with fracture. In a 2021 retrospective study, among a sample of 273 patients, PTH use was found to be associated with lower rates of remote, but not adjacent, fracture [17]. Although this study’s results did not show any significant change in vertebral fracture with Denosumab, a RANKL inhibitor, the sample only included five patients on Denosumab therapy, none of whom experienced an adjacent or remote fracture. Due to our sample size being insufficient to achieve a statistically significant result, larger studies will be required in this area.

Unfortunately, due to the sample size, multiple vertebral-level fracture patterns were not further subclassified and analyzed beyond distinguishing them from single-level injuries.

Numerous other factors have been explored for their possible relationship to AVF following BK. Examples include the time between symptom onset and surgery, with longer intervals being associated with increased AVF incidence [18,19]. One study implicated lower overall rates of AVF in BK performed in the outpatient and ambulatory surgical setting, citing an incidence of 6% [20]. Finally, the severity of fracture based on clinical and radiographic features, as well as pre-operative vertebral instability, have been investigated as associated factors as well [21,22]. These results, taken together, are unfortunately somewhat inconsistent. This is likely due to the considerable heterogeneity of the studies in question and smaller sample sizes. This study adds to the existing body of data and suggests that rates of AVF, 13.5% in this study, are comparable to or lower than values previously reported in the literature for vertebral augmentation procedures.

However, the study has several limitations. Only a retrospective review was performed, with no control comparison available. All patients were drawn from a single center, and all procedures were performed by a single surgeon. Documented histories were unable to be confirmed directly with patients; therefore, issues such as medication adherence could have influenced the data. The onset of the initial fracture was unable to be determined; thus, time from fracture to surgery may be a confounding variable unaccounted for. Finally, the follow-up period was only defined as two months. While this allowed the inclusion of a greater number of patients, it also potentially excluded fractures that occurred later on. However, previous studies have shown the majority of AVF occur within two months [23].

## Conclusions

The treatment of OVCF with balloon kyphoplasty reliably provides symptomatic relief with rates of adjacent refracture comparable to natural history data. The prevalence of refracture may be linked to several modifiable variables yet to be well classified for example sex which this study found a significantly increased risk in male patients, use of concomitant medical therapy which this study found insignificant possibly due to inadequate data, or initial fracture pattern which was unable to be determined in this study. More research is needed to better predict adjacent refracture occurrence in the future and implement reductive strategies.
